# The Present and Future of Optic Pathway Glioma Therapy

**DOI:** 10.3390/cells12192380

**Published:** 2023-09-29

**Authors:** Simone Dal Bello, Deborah Martinuzzi, Yan Tereshko, Daniele Veritti, Valentina Sarao, Gian Luigi Gigli, Paolo Lanzetta, Mariarosaria Valente

**Affiliations:** 1 Clinical Neurology Unit, Santa Maria della Misericordia University Hospital, 33100 Udine, Italy; 2 Department of Medicine—Ophthalmology, University of Udine, 33100 Udine, Italy; 3 Department of Medical Area, University of Udine, 33100 Udine, Italy

**Keywords:** optic pathway glioma, glioma therapy, vision recovery, target therapy, immunomodulation

## Abstract

Optic pathway gliomas (OPGs) encompass two distinct categories: benign pediatric gliomas, which are characterized by favorable prognosis, and malignant adult gliomas, which are aggressive cancers associated with a poor outcome. Our review aims to explore the established standards of care for both types of tumors, highlight the emerging therapeutic strategies for OPG treatment, and propose potential alternative therapies that, while originally studied in a broader glioma context, may hold promise for OPGs pending further investigation. These potential therapies encompass immunotherapy approaches, molecular-targeted therapy, modulation of the tumor microenvironment, nanotechnologies, magnetic hyperthermia therapy, cyberKnife, cannabinoids, and the ketogenic diet. Restoring visual function is a significant challenge in cases where optic nerve damage has occurred due to the tumor or its therapeutic interventions. Numerous approaches, particularly those involving stem cells, are currently being investigated as potential facilitators of visual recovery in these patients.

## 1. Introduction

Optic pathways gliomas (OPGs) fall into two categories: benign gliomas of childhood, a tumor often associated with neurofibromatosis type 1 (NF-1), and malignant glioma [1].

Benign OPGs primarily affect children under 10 years of age; they account for 3 to 5% of childhood tumors of the central nervous system (CNS), whereas they comprise about 1% of all intracranial tumors in the general population [1,2,3]. Benign OPGs of childhood are low-grade neoplasms that originate from precortical optic pathway glial cells and may involve the optic nerve, optic chiasm, optic tracts, optic radiations, or hypothalamus [4]. Histologically, the most frequently encountered tumor is the WHO grade I juvenile pilocytic astrocytoma, although pilomyxoid astrocytomas and grade II diffuse fibrillar astrocytomas have also been reported. [3]. These cancers may occur independently or in association with NF-1 [4]. In NF-1, the neurofibromin, a tumor-suppressor gene located on chromosome 17q, is inactivated, with the subsequent activation of RAS signaling pathways [1,5]. Furthermore, the pathogenetic interaction between NF1-*null* neoplastic astrocytes and NF1-*heterozygous* stromal cells (microglia and endothelial cells) is crucial because the production and modulation of growth factors are essential for glioma formation and growth [6]. When it occurs sporadically, however, the pathogenesis is often related to fusion gene formation involving BRAF and overactivation of RAF/MEK [5]; the most common genetic alteration identified is a BRAF-KIAA1549 fusion [6]. Although histopathological classification is common for these types of tumors, NF-1-associated OPGs are pathogenetically and histologically different from sporadic OPGs [5]. In patients with NF-1, MRI (Magnetic Resonance Imaging) is generally not recommended for the early detection of neoplastic masses, which is performed only if the annual ophthalmologic evaluation shows visual changes. As such, the detection of these gliomas often occurs incidentally [5,7]. Patients may remain asymptomatic or develop various symptoms; the most frequent is a reduced visual acuity that is associated or not with deficits in color perception and/or visual field alteration, but there can also be proptosis and headache [5,8]. Other less frequent symptoms and signs include nystagmus, *spasmus nutans*, seizures, nausea, dizziness, strabismus, developmental regression, growth retardation, and hydrocephalus. These patients may also develop hypothalamic/pituitary dysfunction with precocious puberty, growth hormone deficiency, and deficiency of gonadotropins, TSH, and ACTH [8].

Malignant OPGs, on the other hand, are represented by anaplastic astrocytomas (WHO grade III) or glioblastoma multiforme (WHO grade IV) [7]. These tumors are known to be highly malignant, with a rapid clinical evolution characterized by progressive vision loss, neurological deficits, and eventually death. The age of presentation varies from the second to the eighth decade, and there is no gender-specific predilection [2]. Malignant OPGs are rare tumors characterized by relatively unknown genetic alterations [9].

The increasingly common practice of histological diagnosis through tissue biopsy of OPG, particularly concerning LGG, is becoming a well-established approach [10]. This method allows precise molecular characterization of the tumor, enabling the identification of targeted therapies that can delay or even avoid the need for radiotherapy treatments [10,11]. It is important to underscore, however, that while it can bring significant therapeutic benefits, it also carries noteworthy risks. Possible complications include the emergence of visual deficits, endocrine dysfunctions, hypothalamic disturbances, hemorrhagic events, and, in severe cases, even mortality [10]. Therefore, it is crucial to recognize that, while in the past, this practice was widely employed to confirm a diagnosis, it no longer represents the preferred approach for lesions exhibiting typical imaging features [2,12]. At present, its application is mainly indicated when atypical radiographic characteristics are observed, such as localization outside the optic pathway, peripheral enhancement, the presence of necrotic areas, or diffusion restriction. Furthermore, consideration should also be given in cases where OPG demonstrates clinical or radiographic progression [10,12].

## 2. Standard of Care of Benign Optic Pathways Glioma

The natural history of benign OPGs of childhood is highly variable: some tumors remain stable for years or in some cases, even regress over time, while others may be characterized by progressive growth. At present, treatment is usually reserved for those patients with progressive vision loss or those already suffering from visual impairment who have a high probability of further worsening of vision [3], with or without substantial tumor progression on MRI [13]; therefore, growth in tumor size alone is not used as a criterion for initiating therapy [3]. According to Nicolin et al. [14], approximately 48% of patients do not necessitate therapeutic intervention. Nonetheless, they necessitate neuro-radiological and ophthalmological surveillance, with intervals that vary (quarterly, semi-annually, or annually) contingent upon the tumor’s location, documented symptoms, and the presence or absence of NF-1 association. This monitoring is equally indispensable for cases that have undergone treatment, serving to authenticate treatment effectiveness. During the initial year post-treatment, close monitoring is imperative, after which it may be spaced out if the clinical presentation remains stable.

The potential treatment options currently available include the following: (A) observation, (B) chemotherapy, (C) use of anti-VEGF monoclonal antibodies, (D) radiation therapy, and (E) surgery.

### 2.1. Chemotherapy

In patients with a progressive disease, chemotherapy (CT) is the standard first-line treatment [13]. The combination of **vincristine** and **carboplatin** is the preferred first-line combination therapy; it results in 3- and 5-year progression-free survival (PFS) rates of 77 and 69%, respectively [15]. The TPCV chemotherapy regimen, consisting of **thioguanine**, **procarbazine**, **lomustine**, and **vincristine**, has not shown clear superiority over carboplatin/vincristine in NF-1 patients. Due to the increased risk of hematologic tumors and leukemias associated with lomustine and procarbazine, the TPCV regimen should be avoided for NF-1 patients. However, it may be considered for patients with benign sporadic OPGs [16]. Another chemotherapy combination involves the use of **cisplatin** and **etoposide**, with a 3-year PFS of up to 78% [17]. However, even this regimen should be used with caution given the risk of secondary leukemia with etoposide and of cisplatin ototoxicity. Most recently, **monotherapy** regimens with temozolamide (TMZ), vinblastine, or vinorelbine have also been used, with positive results and low toxicity, even for progressive or refractory disease; however, some studies indicate that temozolamide should be avoided in patients with NF-1 [3]. Chemotherapy has the potential to delay or altogether avoid the use of therapies that may involve potentially greater long-term toxicity or may be demolitive, such as radiation therapy (RT) and surgery. This is especially important for younger patients; indeed, chemotherapy should be the primary treatment modality for OPGs in children younger than 3 years [18]. Visual acuity improvement after chemotherapy is often modest at best. In only 24% of patients with NF-1, visual acuity improves after chemotherapy, while in 35%, it remains stable, and in 41%, it even worsens. Similarly for sporadic gliomas, 18% of patients experience improved vision function, 43% achieves stability, and 39% worsen [19]. However, it must be noted that even this treatment is not without risks, which include renal toxicity, myelosuppression, peripheral neuropathy, ototoxicity, etc. These side effects might be difficult to recognize in very young patients; therefore, strict monitoring with thorough evaluation at regular intervals during and after treatment is required [1]. In addition, posterior tumor location and optic disc abnormality at the initiation of chemotherapy are risk factors for refractory/relapsed NF1-OPG and poor visual outcomes [20].

### 2.2. Anti-VEGF Monoclonal Antibodies

Benign OPGs are often highly vascularized tumors in which increased microvascular density correlates with worse PFS [21]. Vascular endothelial growth factor (VEGF) is abnormally expressed and induces neovascularization in glial neoplasms, including glioblastomas [22]. Bevacizumab is an anti-VEGF monoclonal antibody that, by inhibiting VEGF, is able to reduce tumor growth and vascular permeability [23], thus causing a reduction in tumor volume [24]. Recently, bevacizumab has emerged as a promising treatment for gliomas, including those isolated from the optic nerve (ONGs), either as monotherapy or in combination with irinotecan or other traditional agents. Combination therapy with irinotecan achieved a 2-year PFS of 47.8% in patients with recurrent low-grade gliomas [25,26]. Additionally, in the work of Hwang et al., it was shown that bevacizumab monotherapy does not appear to be less effective than combination treatment, suggesting that monotherapy may be a viable option. Furthermore, bevacizumab therapy also achieved positive responses in improving visual symptoms in up to 86% of refractory cases [27,28]. Considering the results reported in the literature, bevacizumab-based therapy can be used as an option for patients with refractory disease. Bevacizumab is not without side effects, the most common ones being hypertension, fatigue, joint pain, bleeding events, and proteinuria. However, these are generally reversible after the discontinuation of treatment [3].

### 2.3. Radiotherapy

Currently, treating young patients with ionizing radiation is a challenge. It is rarely used in clinical practice because, although it is effective (PFS up to 90% at 10 years), it is burdened by significant side effects [29]. This treatment modality, in fact, can cause serious repercussions on quality of life; it can cause long-term endocrine abnormalities, late cerebrovascular diseases such as Moya Moya syndrome, poor visual outcomes with possible involvement of the contralateral orbit as well, secondary neoplasms, and neurocognitive deficits, especially in young patients [3]. Moreover, it should be considered that nearly 50% of patients with NF-1 who received RT during childhood subsequently developed brain tumors secondary to RT, often high-grade gliomas, which are characterized by a very poor prognosis [30]. For these reasons, radiotherapy has become a last-line therapy and is reserved for patients > 5 years of age who have significant visual or neurological impairment at onset, clinical or radiological progression under close observation, or <5 years of age who progress despite chemotherapy [1]. It has been observed that the efficacy of radiotherapy is maintained whether it is delivered by conventional techniques or by newer methods that minimize the radiation dose that could involve the tissue surrounding the tumor; the latter techniques include stereotaxic radiotherapy, proton beam radiotherapy, and stereotactic radiosurgery (Gamma Knife) [1]. It must be considered, however, that although positive short-term results have been reported, long-term outcomes and analysis of adverse events are still awaiting further studies [3].

### 2.4. Surgery

Resection of OPGs is rarely indicated; new and advanced radiotherapy delivery techniques, which are considered safer and more efficient than surgical treatment, are preferred even in the most severe cases. Surgical removal of the lesion through an orbital approach and/or craniotomy should therefore be considered only in the presence of painful or disfiguring proptosis, exposure keratopathy in a severely visually impaired eye, or radiologically documented tumor enlargement or extension (not involving the optic chiasm), or a combination of these [1,3]. Combined intracranial and intraorbital surgery carries a high risk of visual, endocrinological, and cerebrovascular morbidity [3].

## 3. Standard of Care of Malignant Optic Pathways Glioma

Malignant OPGs are a rare and fatal disease that predominantly affects the adult population and for which there is currently no therapy that can halt its growth. Although encouraging short-term results have been initially observed with the combination of temozolomide chemotherapy and radiation, the treatment remains unsatisfactory, similar to that for other glioblastomas (GBMs) [1,31]. Indeed, with the exception of adjunctive chemotherapy regimens, including TMZ and bevacizumab, most chemotherapeutic agents show limited efficacy in GBM therapy due to poor solubility, rapid degradation and clearance, insufficient tumor uptake, and a lack of selectivity associated with intolerable adverse effects. As mentioned earlier, the blood–brain barrier (BBB) further limits drug delivery to the brain, posing another challenge and limiting therapeutic options [32]. Given the limited efficacy of current treatments, a notable subset of GBM patients necessitate a secondary treatment line. This may encompass further surgical resection, additional radiotherapy, systemic therapies such as lomustine or bevacizumab, combined therapeutic approaches, or the provision of supportive care [33].

## 4. New Therapeutic Perspectives

In recent years, studies have been conducted in order to expand the therapeutic range for OPGs, both malignant and benign. Indeed, the rationale in the former case is to identify therapeutic protocols that can improve the prognosis for these patients, which is currently very poor; in the latter case, on the other hand, efforts are being made not only to find less invasive and even more effective therapies but also to recover at least part of the vision that is lost in the course of the disease and its treatment. A review of the literature and ongoing clinical trials reveals a restricted corpus of research dedicated to the investigation of innovative therapeutic options for OPGs. In contrast, there are significantly more studies conducted on gliomas in general. Table 1 provides an overview of clinical studies investigating the potential application of new therapies in OPGs.

As mentioned above, the pathogenesis of NF-1-associated OPGs involves the constitutive activation of RAS, resulting in the promotion of cell growth and proliferation. These pro-oncogenic properties of the RAS protein depend on its ability to activate the RAS/MEK/ERK and the AKT/mTOR pathways, resulting in the reduction of intracytoplasmic cAMP levels in astrocytes [5]. In consideration of this, MEK inhibitors such as **selumetinib** and **trametinib** have recently been used in the treatment of progressive and recurrent low-grade gliomas in children, demonstrating a 2-year PFS of up to 69% [34]; their efficacy is likely to be greatest in patients with BRAF V600 mutations. Moreover, the utilization of selumetinib is linked to a prolonged disease stability [35]; in fact, its application as an alternative to standard chemotherapy has been hypothesized [36]. An ongoing phase III study aims to juxtapose the efficacy of selumetinib with carboplatin/vincristine-based chemotherapy. It must be specified, however, that selumetinib has been associated with ocular adverse effects, although they may be reversible in younger patients. Cases of optic neuropathy, retinal vein occlusion, uveitis, sensorineural retinal detachment, and retinopathy associated with MEK inhibitors have been reported in the adult population. Molecularly targeted combination therapies are under investigation, but MEK inhibitors are currently a viable option for salvage treatment in both sporadic and NF-1-associated OPGs [3]. Another promising drug appears to be **lenalidomide**. It has demonstrated adequate activity in children with LGG, even at low doses, warranting further extensive future studies [37]. A phase II study is also underway to investigate the potential use of **Pegylated Interferon α-2b** in juvenile pilocytic astrocytomas and OPGs. This drug has also been explored as a potential therapeutic option for diffuse intrinsic pontine glioma and recurrent glioblastoma multiforme; however, it is not able to increase the 2-year survival, although it has been shown to extend the tumor progression time [38] and enhance therapeutic effectiveness when combined with TMZ compared to the latter in monotherapy [39]. Finally, there are two ongoing studies on Entinostat and Irinotecan and a study on the use of proton therapy, as it has shown the capability to reduce the radiation dose to the brain, thus decreasing acute toxicity without compromising disease control [40].

**Table 1:** Overview of clinical studies investigating the potential application of new therapies in OPGs.

Drug Name	Mechanism of Action	Tumor Type	Results	References
Selumetinib	MEK inhibitor	LGG, including progressive, recurrent or refractory optic pathway glioma Progressive/relapse OPGSs or inoperable plexiform neurofibromas LGG and OPGs	Phase II study showing potential clinical benefit Ongoing Phase I anc II studies Ongoing phase III study	[36] Clinicaltrials.gov: Intermittent Dosing Of Selumetinib In Childhood NF1 Associated Tumours NCT03326388 Clinicaltrials.gov: A Study of the Drugs Selumetinib Versus Carboplatin/Vincristine in Patients With Neurofibromatosis and Low-Grade Glioma. NCT03871257
Trametinib	MEK inhibitor	Pediatric Neuro-oncology Patients with Refractory Tumor and Activation of the MAPK/ERK Pathway	Ongoing phase II study	Clinicaltrials.gov: Trametinib for Pediatric Neuro-oncology Patients With Refractory Tumor and Activation of the MAPK/ERK Pathway. NCT03363217
Lenalidomide	It induces ubiquitination and degradation of the lymphoid transcription factors Ikaros (IKZF1) and Aiolos (IKZF3) via the cereblon (CRBN) E3 ubiquitin ligase for proteasomal degradation	Progressive, recurrent or refractory optic pathway glioma	Phase II study showing potential clinical benefit	[37]
Pegylated interferon alfa-2b (PEG-Intron)	It binds to and activates human type 1 interferon receptors causing them to dimerize. This activates the JAK/STAT pathway. Peginterferon alfa-2b may also acitvate the nuclear factor κB pathway.	Juvenile pilocytic astrocytomas or optic patheway gliomas.	Ongoing Phase II Study	Clinicaltrials.gov: Pegylated Interferon ALFA-2b in Children With Juvenile Pilocytic Astrocytomas and Optic Pathway Gliomas. NCT02343224
Entinostat	Benzamide histone deacetylase inhibitor	Pediatric patients with recurrent or refractory solid tumors Pediatric patients with recurrent or refractory solid tumors	Phase I study concluded, showing good safety and tolerance Ongoing phase I study	[41] Clinicaltrials.gov: Entinostat in Treating Pediatric Patients With Recurrent or Refractory Solid Tumors. NCT02780804
Irinotecan	It is a prodrug that undergoes de-esterification to the more potent topoisomerase I inhibitor, SN-38	Children with refractory solid tumors Children with refractory solid tumors	Phase II study in monotherapy showing no clinical benefit Ongoing phase II study	[42] Clinicaltrials.gov: Irinotecan in Treating Children With Refractory Solid Tumors NCT00004078
Proton therapy	Physical therapy	LGG including OPGs	Ongoing Phase II Study	Clinicaltrials.gov: Evaluation of Hippocampal-Avoidance Using Proton Therapy in Low-Grade Glioma NCT04065776

Considering the limited available literature on novel therapeutic approaches specifically for OPGs, we have explored treatments that have been evaluated for gliomas in general and which, in our view, could potentially be applicable in OPGs after appropriate studies.

### 4.1. Immunotherapy

Tumor cells accumulate mutations during their development, which should trigger an immune response against them. However, cancer cells employ various mechanisms to evade the immune response, such as impairing antigen presentation and activating immunosuppressive pathways [43]. Immunotherapy aims to restore the immune response against cancer cells [44].

A new therapeutic possibility Involves the utilization of **oncolytic viruses**. Oncolytic viruses can selectively infect tumor cells due to the presence of specific receptors on the cell surface and the absence of an Interferon-1-mediated response or resistance of the virus itself to the antiviral effects inducible by interferon [45,46]. Some mouse studies have been conducted on the Nf1;Trp53 mutant astrocytoma/glioblastoma model, which show that tumor cells are susceptible to viral infection and replication in culture but are resistant when implanted into immunocompetent mice [44,47]. In addition, the death of cancer cells results in their release into the extracellular environment of proinflammatory cytokines that can activate the tumor-specific immune response [45]. Various oncolytic viruses are in development; among them are tasadenoturev (derived from human adenovirus type 5) [48], Newcaste disease virus [49], myxoma virus [50], Semliki forest virus [51], measles virus [52], reovirus [53], parvovirus H-1 [54], M1 oncolytic virus [55], recombinant vesicular stomatitis virus and double-deletion vaccination virus [56], Herpes Simplex virus [57], and Zika virus [58]. At present, the specific mechanism of action and side effects of some viruses are still under study. As for gliomas, the oncolytic virus that could play a role in future therapeutic strategies is the myxoma virus, which can induce apoptosis in malignant gliomas by activating AKT and increasing the amount of phosphorylated AKT [45].

Another therapeutic technology capable of inducing apoptosis in cancer cells is represented by **Car-T cells**. Unfortunately, Car-T cells have shown limited efficacy against brain tumors due to the presence of the BBB, which, although heterogeneously disrupted, limits the entry of these cells into the site, heterogeneity in target antigen expression, and the immunosuppressive tumor microenvironment [59]. To resolve these obstacles and improve efficacy, next-generation Car-Ts have been developed. Gene editing allows the overexpression of cytokines, gene knock-out and knock-in, simultaneous targeting of multiple antigens, and precise control of CAR expression and signaling. These next-generation Car-T cells have shown promising results in preclinical models and could be the key to harnessing the full potential of Car-T cells in the treatment of high-grade glioma (HGG) [60].

Additionally, it has been hypothesized that, to enhance the efficacy of Car-T, oncolytic viruses could be used for the treatment of glioma in immunocompetent murine models. This treatment has led to prolonged survival in mice with gliomas. Stimulation of the native T cell receptor (TCR) with viral or virally encoded epitopes results in increased proliferation, direct antitumor function of Car-T cells, and distinct memory phenotypes [61].

Another innovative therapy under investigation is anti-tumor vaccination, which aims to activate immunity against glioma by injecting tumor components [44]. Currently, trials are under way on various types of anti-glioma/glioblastoma vaccines, such as the dendritic cell vaccine. Some types of these vaccines are composed of antigen-presenting cells activated by autologous or allogeneic tumor lysate, tumor-associated antigenic peptides, or transinfected by mRNA. Other vaccines, however, consist of inactivated autologous glioma cells mixed with GM-CSF-producing bystander cells [62,63]. These vaccines are currently promising for pediatric low-grade glioma (LGG) [44].

Lastly, we have synthesized in Table 2 the recently developed and/or investigational immunotherapeutic drugs that could potentially play a role in OPG therapy.

Tumors with a high number of mutations are expected to exhibit heightened responsiveness to immunotherapies [64]. Nonetheless, concerning GBMs, limited success has been observed, with the most promising outcomes associated with **Pembrolizumab** (anti-PD-1) [44] employed as neoadjuvant therapy [65] and **Ipilimumab** (anti-CTLA-4) used in combination with other immunotherapeutic modalities [66,67]. Also under investigation is the potential utilization of an **antibody targeted against HAVCR-2** (Hepatitis A Virus Cellular Receptor 2), also known as TIM-3 [68]. It participates in immunological tolerance and inhibition of Th1 responses in gliomas, synergistically with PD-L1 [68,69,70]. Promising efficacy has been reported in animal models using a combination of anti-HAVCR2 antibody therapy, anti-PD-1 therapy, and radiotherapy [71].

Therapy using cytokines makes it possible to directly stimulate the growth and activation of cells in the immune system [72]. Multiple cytokines, such as interleukins and interferons, have already been widely used in the therapy of a variety of cancers, often in combination with other therapies [62]. Indeed, **tocilizumab**, a humanized antibody targeting the IL-6 receptor, has proven effective in an in vivo xenograft model of GBM, especially in combination with TMZ, due to its promotion of a direct immune response against the tumor and induction of apoptosis in cancer cells [44,73]. Other therapeutic options, still aimed at enhancing the direct immune response against the tumor, are under investigation. For instance, an ongoing phase I clinical trial is exploring the potential utilization of an **antibody targeting LAG-3** (Lymphocyte Activation Gene 3) [62]. Because this protein is overexpressed in T cells with a loss of function within the tumor tissue of GBM and it triggers an immune evasion like that mediated by PD-1, it could be a future therapeutic option for this tumor [74].

Also related to GBM, the possible use of a nasal administration-mixed cationic nanoemulsion based on **CD73-siRNA** is being investigated [75]; CD73 is an extracellular nucleotidase that exerts immunosuppressive effects and induces drug resistance in GBM [76]. In studies conducted on animals, promising results have been observed in reducing tumor volume, as it has been shown to decrease tumor volume by 60%, and increase cancer cell sensitivity to chemotherapy [75]. Indoleamine 2,3-dioxygenase 1 (IDO1) also promotes immunosuppression and immunotolerance in gliomas, as well as increasing their malignancy [77]. Inhibiting it with the drug **Indoximod**, in combination with TMZ, has shown efficacy in animal models of grade IV glioma [78], and it is currently under investigation in a phase II study. CD70 is a receptor that is overexpressed in glial tumor cells associated with poor survival [79], which binds CD27 [80]. Because CD27 induces the cytotoxicity of CD27-transporting lymphocytes, the possible use of its inhibitor (**Varlilumab**) is being investigated as neoadjuvant therapy or in combination with other immunotherapy drugs [81]. Additional studies are underway: a phase I/II study of **Mebendazole** in combination with standard-of-care agents [82] and a study about **PTC299**, which can stop the growth of tumor cells by blocking blood flow to the tumor [83].

**Table 2:** Recently developed and/or investigational immunotherapeutic drugs that could potentially play a role in OPG therapy.

Drug Name	Mechanism of Action	Tumor Type	Results	References
Pembrolizumab	Anti-PD-1 monoclonal antibody	Recurrent Glioblastoma	Phase II study showing potential clinical benefit of neoadjuvant use	[65]
Ipilimumab	Anti-CTLA-4 monoclonal antibody	Recurrent Glioblastoma	Phase I study concluded, showing good safety and tolerance	[84]
Atezolizumab	Anti-PD-L1 monoclonal antibody	Recurrent Glioblastoma	Phase I study concluded, showing good safety and tolerance	[85]
Tocilizumab	Anti-IL-6 receptor monoclonal antibody	Glioblastoma	Pre-clinical study demonstrating therapeutic efficacy in a xenograft model in vivo of malignant glioma, especially in combination with temozolomide.	[73]
BMS-986016	Anti-LAG-3 monoclonal antibody	Advance solid tumors	Ongoing Phase I Study	Clinicaltrials.gov: Safety Study of BMS-986016 With or Without Nivolumab in Patients With Advanced Solid Tumors. NCT02966548
MBG-453	Anti-HAVCR2 monoclonal antibody	Recurrent glioblastoma	Ongoing phase I study in combination therapy	Clinicaltrials.gov: Trial of Anti-Tim-3 in Combination With Anti-PD-1 and SRS in Recurrent GBM. NCT03961971
NE-siRNA CD73R	CD73-siRNA	Glioblastoma	Pre-clinical in vitro and mouse model studies in which therapeutic potential is shown	[75]
Indoximod	Inhibition of Indoleamine 2,3-dioxygenase 1	Children with relapsed brain tumors or newly diagnosed DIPG	Ongoing phase II study in combination therapy	Clinicaltrials.gov: Pediatric Trial of Indoximod With Chemotherapy and Radiation for Relapsed Brain Tumors or Newly Diagnosed DIPG. NCT04049669
Varlilumab	Anti-CD27 monoclonal antibody	Advanced Refractory Solid Tumors Low Grade Glioma Newly diagnosed glioblastoma	Phase I and II studies concluded, showing good safety and tolerance but no clinical benefit Ongoing phase I study in combination therapy Ongoing phase II study in combination therapy	[86] Clinicaltrials.gov: A Study of Varlilumab and IMA950 Vaccine Plus Poly-ICLC in Patients With WHO Grade II Low-Grade Glioma (LGG). NCT02924038 Clinicaltrials.gov: DC Migration Study to Evaluate TReg Depletion In GBM Patients With and Without Varlilumab (DERIVe). NCT03688178
Mebendazole	It inhibits both microtubule formation and glucose uptake. Although mebendazole can interfere with several key oncogenic signal transduction pathways	Pediatric glioma	Ongoing phase I and II studies in combination therapy	Clinicaltrials.gov: A Phase I Study of Mebendazole for the Treatment of Pediatric Glioma. NCT01837862
PTC299	It selectively inhibits vascular endothelial growth factor receptor protein synthesis at the post-transcriptional level	Pediatric patients with refractory or recurrent CNS tumors	Phase I study concluded, showing good safety and tolerance	[83]

### 4.2. Molecular-Targeted Therapy for NF-1 Associated Tumors

**Ketotifen** is a mast cell stabilizer known for its ability to block cytokine secretion. In some case reports, it has been used for prevention purposes in NF-1 [44]. **Imatinib** is a powerful multityrosine kinase inhibitor that targets c-Kit, PDGFR, and ABL. It has demonstrated efficacy in blocking mast cell proliferation (through c-KIT inhibition), inhibiting neoplastic cell growth, and suppressing angiogenesis by targeting PDGFR [44]. Imatinib has also shown the potential to enhance apoptosis in neoplastic cells and increase sensitivity to irinotecan [87]. In both pediatric and adult forms of glioma, glioblastoma, and myxoid glioneuronal tumors, somatic alterations of PDGFR have been observed, making imatinib a promising therapeutic candidate [44,88]. Also, **Sunitinib**, another multityrosine kinase inhibitor, exhibits activity against VEGFR, PDGFR, and c-KIT [44]. Like imatinib, it can impede the growth of neurofibromas by employing multiple mechanisms. Sunitinib has demonstrated antiangiogenic effects on murine gliomas and exhibits various other off-target effects. However, its efficacy does not appear to surpass that of other studied therapies [89]. **Pazopanib**, a multitarget tyrosine kinase inhibitor, directed against VEGFR-1, -2, and -3, platelet-α- and -β-derived growth factor receptors, and c-Kit, is being studied in rGBM [90,91]. **Sulindac** and **Celecoxib** are nonsteroidal anti-inflammatory drugs that inhibit various biological functions through both COX1/2-dependent and independent mechanisms. In vitro studies have shown that these drugs effectively inhibit GBM proliferation [92]. Sulindac inhibits the bioactivity of IL-6, TGF-α, and TGF-β, while also reducing the number of macrophages in the tumor microenvironment [44]. Additionally, both sulindac and celecoxib can activate caspases, inducing apoptosis. Sulindac, with its good brain bioavailability, lower COX-2 inhibition, and lack of mitochondrial effects, emerges as a more appealing candidate than celecoxib for achieving gliotoxicity [92].

### 4.3. Tumor Microenviroment

Gliomas represent complex and heterogeneous cellular ecosystems, where different cell types, including non-neoplastic cells, contribute to the maintenance and progression of the tumor [93]. Recognizing the crucial role of the tumor microenvironment in the pathogenesis of LGG, novel therapeutic strategies aim to disrupt the interactions between neoplastic cells and the various cell populations within the microenvironment. By targeting these interactions, these therapies strive to undermine the supportive network that fuels tumor growth and development [94,95].

Approximately 35–50% of the cellular composition of NF-1-associated gliomas consists of non-neoplastic stromal cells, mainly microglia, which are a population of monocytes residing in the CNS [5,96]. Studies have demonstrated that the development of OPGs is preceded by an increase in astrocyte proliferation and an augmentation of microglia cells in the tumor area [5,97]. In fact, it has been demonstrated that the emergence and growth of OPGs rely on microglia, particularly on the growth factor Ccl5 produced by these cells [93]. In addition, the loss of the second Nf1 allele in astroglial progenitors alone is insufficient for tumor development: the interaction with the microenvironment is also crucial [5,97]. These cellular changes, coupled with apoptosis of retinal ganglion cells (RGCs) and structural disarray of the optic nerve, precede the detectable appearance of the tumor through imaging techniques [97]. This insight opens possibilities for therapies that target not only the tumor cells, but also the milieu in which they thrive. A genetic reduction in microglia recruitment, identified by ITGAL/CD11A markers [96], has been shown to delay tumor onset. Furthermore, pharmacological inhibition of microglia and of the cytokines produced by them has demonstrated efficacy in reducing tumor growth and proliferation in vivo [94,95,98]. Antibodies directed against CD-11a have also been shown to decrease glioma proliferation and volume in in vivo studies [96].

Beyond microglia, which contribute to tumor formation and growth by processing growth factors and chemokines [95,97], it is postulated that other internal and external factors may influence tumor growth such as the promotion of synaptogenic-related growth, or even light exposure. Intriguingly, studies have shown that NF-1-mutated mice exposed to a dark environment during the period of glioma development exhibit tumors with a lower level of proliferation [99].

Compared to the normal cellular environment, gliomas exhibit a higher presence of cytotoxic T cells, which interact with microglia and growth factors, playing a role in tumor proliferation [44,93]. Notably, mutant neurons in NF-1 have been found to produce midkine, which activates T cells, which secrete Ccl4, inducing the production of Ccl5 by microglia to sustain the growth of LGG cells. The inhibition of integrin-mediated T cell entry has been shown to effectively attenuate tumor growth in vivo. Hence, the use of antibodies targeting VLA4, CD3, and CD8 has also been proposed as a potential strategy to reduce recruitment and activation of T lymphocytes, thereby limiting LGG growth [93].

Tumor-associated macrophages (TAMs) constitute a significant cellular component within the tumor microenvironment, exerting regulatory functions that facilitate tumor progression. Notably, the abundance of M2-polarized TAMs, characterized by their immunosuppressive phenotype, has been observed to correlate with tumor histologic grade. Given the critical role of M-CSF in the polarization of microglia/macrophages toward the M2 subtype, the inhibition of M-CSF has shown efficacy in murine models of GBM, leading to improved overall survival [62].

### 4.4. New Support Approaches

The administration of chemotherapeutic agents is often limited by various pharmacokinetic challenges. Extensive research efforts are focused on developing nanotechnological solutions, such as the formulation of nanoparticle systems including liposomes, polymeric micelles, and inorganic nanocarriers. These nanocarriers enable the efficient delivery of chemotherapeutic drugs, antibodies, or nucleic acids, thereby improving their bioavailability, stability, and solubility. Additionally, through passive or active targeting strategies, these nanotherapies can enhance the accumulation and retention of therapeutic payloads, specifically at the GBM site. Consequently, these approaches have substantial potential for minimizing systemic toxicity associated with chemotherapy [32].

The CyberKnife system, a frameless image-guided robotic radiosurgery modality, allows for the delivery of carefully targeted radiation beams to the tumor with submillimeter precision, thereby minimizing the doses applied to surrounding healthy tissue. CyberKnife can also continuously monitor tumor displacement, correcting errors in real time [100]. Such treatment in the future could be very useful for treating gliomas/glioblastomas of the optic pathways, allowing it to selectively target the tumor mass while minimizing damage to the visual pathways [100].

Hyperthermia therapy (HT) is a treatment modality that involves raising the temperature of a specific region or the entire body above normal levels. Magnetic hyperthermia therapy (MHT) is a specific form of HT that has shown efficacy in the treatment of various cancer types. Preliminary results indicate that MHT confers a significant antitumor effect and positively influences the overall survival in glioma patients. However, further advancements in MHT technology are needed to fully realize its potential and facilitate its integration into future brain cancer treatment approaches [101].

Emerging research has revealed that HGG possesses components of the endocannabinoid system, such as CB1 and CB1 receptors for cannabinoids. Their pharmacological activation on glioma cells has demonstrated potent antitumor effects in preclinical studies. The underlying mechanism, although complex and not fully elucidated, involves the modulation of crucial intracellular signaling pathways. This activation not only inhibits tumor cell survival and proliferation but also disrupts the invasiveness, angiogenesis, and stem cell-like properties of cancer cells [102].

Recent scientific interest has emerged regarding the use of the ketogenic diet (KD) as a complementary therapy for various neoplasms, including those of the CNS. The KD facilitates metabolic substrates for normal cells while simultaneously orchestrating selective tumor cytotoxicity via diverse pathways encompassing metabolic modulation, inflammation attenuation, oncosuppressor pathway activation, oncogene blockade, and epigenetic target modulation. Advantages of the KD include low toxicity, affordability, and ease of implementation. However, patient adherence poses a significant challenge. Further clinical trials are needed to obtain conclusive data on the effectiveness of the KD in CNS tumors. Future prospects may encompass the integration of the KD with traditional chemotherapeutic and radiotherapeutic strategies [103].

A pilot study is underway on the use of **MRI-guided laser thermal ablation** to induce peritumoral BBB disruption to improve the delivery and treatment efficacy of pediatric brain tumors [104].

Drug trials are underway on the use of **radiolabeled monoclonal antibodies**, such as the monoclonal antibody iodine I 131 3F8, against CNS tumors [105]. These antibodies may selectively identify and bind tumor cells, thereby delivering cytotoxic agents that specifically target neoplastic cells, sparing healthy cells.

**Photodynamic therapy** (PDT), a novel therapeutic approach under investigation for the treatment of gliomas and glioblastomas, is also noteworthy [106]. PDT involves the photo-activation of a photosensitizer molecule that is selectively incorporated into neoplastic cells, thus enabling targeted destruction of the tumor [107].

## 5. Potential Strategies for Vision Recovery

Visual decline serves as a prominent criterion for determining the optimal timing of treatment initiation in OPGs. However, it is critical to be able to identify the predictive factors of visual decline in order to anticipate the clinical evolution and intervene therapeutically before there is a significant visual impairment [108]. Several studies have endeavored to establish a therapeutic window that exploits the lag between the onset of RGC loss and the initial manifestation of declining visual acuity, typically occurring after a 50% reduction in RGC count [109]. Diagnostic modalities employed in this context include optical coherence tomography (OCT) for measuring the retinal nerve fiber layer (RNFL) and visual evoked potentials (VEPs) [108]. Neuro-ophthalmological manifestations depend upon tumor localization, such as vision loss due to monocular or bilateral optic neuropathy when the optic nerves are involved, bitemporal hemianopsia in cases involving the optic chiasm, or homonymous hemianopsia in retrochiasmatic location [110]. Evaluating and quantifying visual acuity in children with these tumors proves challenging, given the frequent comorbidities of attention deficit, hyperactivity, or behavioral disorders [109]. Visual impairment in optic nerve gliomas (ONGs), particularly in the presence of NF-1, stems from retrograde axonal degeneration. Inflammatory processes, microglial activation, heightened cytokine production, and morphological alterations contribute to axonal damage, which is subsequently transmitted to the cell body of the RGCs [97,109,111]. Indeed, in engineered mouse models carrying NF-1, tumorigenesis is accompanied by cell death and impaired axonal function of RGCs, as evidenced by the thinning of RNFL [95,109]. However, it is important to note that visual decline is not only limited to the active disease phase. In fact, even after therapeutic administration and tumor mass reduction through chemotherapy, visual deficits persist in approximately 30% of children. Unfortunately, specific therapies for the restoration of visual loss in these cases are currently unavailable [5,112]. Neuronal growth factor (NGF) may play a role in this context, as it is a neurotrophic factor crucial for the development and survival of neurons [112], offering neuroprotective properties [95]. In animal models, NGF has demonstrated its ability to facilitate axonal and neuronal growth in response to ischemic, toxic, or inflammatory injuries to nerve tissue [112]. Notably, other growth factors have shown potential in inducing optic nerve regeneration in adult mammals when administered via the intraocular route [111]. In addition, given the presence of NGF receptors on RGCs, as well as on conjunctiva and cornea, topical application through eye drops has been suggested as an alternative delivery method [112]. Activation of the TrkA receptor on RGCs by NGF triggers upregulation of the anti-apoptotic protein Bcl-2, inhibiting caspase activation and subsequent cell apoptosis. Furthermore, NGF exhibits neosynaptogenic effects and stimulates the production of additional growth factors, including BDNF, which contribute to neuroprotection [112]. Importantly, treatments promoting axonal regeneration have been found to prevent RGC death, suggesting a correlation between cell survival and axonal growth [111]. However, caution is warranted when considering this therapeutic approach, as it has the potential to also increase tumor volume [112]. Additionally, it is unclear whether the newly formed axons can establish appropriate connections within the adult brain, avoiding the formation of aberrant connections and patterns [111]. To enhance visual outcomes, some authors have hypothesized that RGC survival can be increased by raising cAMP levels or reducing microglia-associated tumor-induced axonal damage. Specifically, this approach may involve interfering with Erβ-mediated microglia reprogramming or disrupting paracrine signaling pathways implicated in axonal damage and apoptosis [95]. Notably, studies have shown increased RGC survival with the administration of rolipram, an inhibitor of cAMP degradation, supporting this hypothesis [109]. Considering the detrimental effects of OPG on the ganglion cell layer, exploring cell replacement strategies emerges as a promising therapeutic approach to restore the lost cells and rehabilitate visual acuity or visual field. The aim is to induce cellular differentiation towards RGCs and subsequent integration at the retinal level, either through the differentiation of the patient’s own embryonic or pluripotent stem cells or by utilizing adult fibroblasts [111]. Reprogramming adult fibroblasts into pluripotent stem cells via the activation of specific transcription factors offers a potential approach for subsequent RGC differentiation. However, a major challenge lies in facilitating the appropriate engraftment of these cells at the retinal level and promoting the growth of axons capable of reaching the cortex. Experimental studies have revealed that less than 20% of intravitreally injected cells successfully integrate at the retinal level, and merely around 10% of these are able to develop axons long enough to cross the lamina cribrosa. The limited axonogenesis observed within the CNS may be attributed to the absence of external guidance cues or the presence of inhibitory molecules. Electric fields have demonstrated potential in guiding the directional growth of RGC axons, as evidenced by in vitro studies. Additionally, several outstanding questions persist, such as whether the regenerated axons will establish functional synapses with the diencephalon while preserving the retinotopic map and whether myelination will occur. Alternative approaches to promoting synaptogenesis may exploit mechanisms involved in developmental synapse formation. For instance, the combination of high-contrast visual stimulation with mTOR activation has exhibited enhanced RGC regeneration and partial restoration of visual function following crush injuries [113].

## 6. Conclusions

Malignant OPGs are cancers that are characterized by high mortality and for which, currently, there are no therapies that can guarantee a satisfactory prognosis. For this reason, several studies are underway with the aim of finding new therapeutic approaches that can increase overall survival.

For benign OPGs, on the other hand, standard therapies already provide a good prognosis. The main goal in this case is to identify new therapeutic approaches to decrease adverse effects in both the short and long term. In particular, several studies are searching for possible methods to restore at least some of the vision lost in the course of the disease or its treatment.

## Data Availability

Not applicable.

## Abbreviations

BBB	Blood–brain barrier
BDNF	Brain-derived neurotropic factor
cAMP	Cyclic adenosine monophosphate
CAR	Chimeric antigen receptor
CNS	Central nervous system
COX	Cyclooxygenase
CT	Chemotherapy
CTLA-4	Cytotoxic T-lymphocyte-associated protein 4
GBM	Glioblastoma
GM-CSF	Granulocyte-macrophage colony-stimulating factor
HAVCR2	Hepatitis A virus cellular receptor 2
HGG	High-grade glioma
HT	Hyperthermia therapy
IDO1	Indoleamine 2,3-dioxygenase 1
IFN	Interferon
IL	Interleukin
KD	Ketogenic diet
LAG-3	Lymphocyte activation gene 3
LLG	Low-grade glioma
MAP	Mitogen-activated protein
M-CSF	Macrophage colony-stimulating factor
MHT	Magnetic hyperthermia therapy
MRI	Magnetic Resonance Imaging
mRNA	Messenger Ribonucleic Acid
NF-1	Neurofibromatosis type 1
NGF	Neuronal growth factor
NPs	Nanoparticles
OCT	Optical coherence tomography
ONGs	Optic nerve gliomas
OPGs	Optic pathway gliomas
PBCT	Proton boron capture therapy
PD-1	Programmed cell death protein 1
PDGFR	Platelet-derived growth factor receptor
PD-L1	Programmed death-ligand 1
PDT	Photodynamic therapy
PFS	Progression free survival
PT	Proton therapy
rGBM	Recurrent glioblastoma
RGCs	Retinal ganglion cells
RNFL	Retinal nerve fiber layer
RT	Radiotherapy
siRNA	Small interfering RNA
TAMs	Tumor-associated macrophages
TCR	T cell receptor
TGF	Transforming growth factor
TMZ	Temozolamide
TNF	Tumor necrosis factor
TNFR	Tumor necrosis factor receptor
TTFields	Tumor-treating fields
VEGF	Vascular endothelial growth factor
VEPs	Visual evoked potentials
VLA-4	Very late antigen 4
WHO	World Health Organization
